# Exploring antenatal education content for couples in Blantyre, Malawi

**DOI:** 10.1186/s12884-018-2137-y

**Published:** 2018-12-17

**Authors:** Maria Chifuniro Chikalipo, Ellen Mbweza Chirwa, Adamson Sinjani Muula

**Affiliations:** 1 School of Public Health and Family Medicine, College of Medicine, University of Malawi, Blantyre, Malawi; 2 Kamuzu College of Nursing, University of Malawi, Blantyre, Malawi; 3 Africa Center of Excellence in Public Health and Herbal Medicine, University of Malawi, Blantyre, Malawi

**Keywords:** Antenatal education, Childbirth preparation, Parenthood, Information and antenatal education, Expectant couple and childbirth

## Abstract

**Background:**

Despite advocating for male involvement in antenatal education, there is unmet need for antenatal education information for expectant couples. The objective of this study was to gain a deeper understanding of the education content for couples during antenatal education sessions in Malawi. This is needed for the development of a tailor-made curriculum for couple antenatal education in the country, later to be tested for acceptability, feasibility and effectiveness.

**Methods:**

An exploratory cross sectional descriptive study using a qualitative approach was conducted in semi-urban areas of Blantyre District in Malawi from February to August 2016. We conducted four focus group discussions (FGDs) among men and women independently. We also conducted one focus group discussion with nurses/ midwives, 13 key informant interviews whose participants were drawn from both health-related and non-health related institutions; 10 in-depth interviews with couples and 10 separate in-depth interviews with men who had attended antenatal clinics before with their spouses. All the interviews were audiotaped, transcribed verbatim and translated from Chichewa, the local language, into English. We managed data with NVivo 10.0 and used the thematic content approach as a guide for analysis.

**Results:**

We identified one overarching theme: couple antenatal education information needs. The theme had three subthemes which were identified based on the three domains of the maternity cycle which are pregnancy, labour and delivery and postpartum period. Preferred topics were; description of pregnancy, care of pregnant women, role of men during perinatal period, family life birth preparedness and complication readiness plan, coitus during pregnancy and after delivery, childbirth and baby care.

**Conclusion:**

Antenatal education is a potential platform to disseminate information and discuss with male partners the childbearing period and early parenting. Hence, if both men and women were to participate in antenatal education, their information needs should be prioritized. Men and women had similar choices of topics to be taught during couple antenatal education, with some minor variations.

**Electronic supplementary material:**

The online version of this article (10.1186/s12884-018-2137-y) contains supplementary material, which is available to authorized users.

## Background information

Antenatal education is one of the pillars of antenatal care which aims at improving the health of mothers, babies and their families. Through health information obtained from antenatal education sessions, women and their families are prepared for pregnancy, childbirth and parenthood [1, 2]. Additionally, in low and middle-income countries where traditional beliefs may be more important, antenatal education can be used as a tool to dispel myths associated with child birth [3–5].

Globally, antenatal education has focused on women; and recently male involvement in maternal health services including antenatal education has been advocated [6–10]. This argument is supported by the rationale that men are likely to participate in maternal and child health issues and fulfill their supportive roles as husbands and partners if they are knowledgeable about pregnancy, childbirth and early parenting. Despite this recognition, evidence suggests that there is suboptimal information within antenatal education for expectant fathers [8, 11–13]. Furthermore the information that these fathers need to know remains unclear [14].

While studies have been conducted on antenatal education, the focus has been on information needs for women and only a few on male partners and couples; with the majority of the studies done in high-income countries. Although preferred content for antenatal education may vary within and between regions, reported topics of interest for women globally include pregnancy and its care; labour, delivery and postpartum care of the mother and baby; role of women during the perinatal period; and psychosocial aspects of pregnancy [15–18]. Similarly, studies in low and middle-income countries have documented pregnancy, labour and delivery, postpartum care for the mother and baby, relationships, sex during and after birth, communication and the role of men during the perinatal period as preferred topics to be taught to men and women during antenatal education [7, 15, 19–21]. However, in Sweden women wanted more information on postpartum needs such as breastfeeding problems, while men’s information needs were baby care skills, sexuality and relationships [22–24]. In contrast, in Nigeria, women wanted their spouses to learn about the effects of pregnancy on the woman, how to care for a pregnant woman, how to be patient and understanding with their partner and; sex during pregnancy [25].

It appears few studies have been conducted about the information needs of expectant father [4, 26, 27], particularly in low and middle-income countries where male involvement in maternal health is an emerging phenomenon. To ensure the relevance and importance of antenatal education classes, in light of male involvement, an assessment of men’s needs in terms of topics is required [14]. In support of this view, evidence further shows that basing instructional content on needs of programme beneficiaries may lead to the development of appropriate and culturally acceptable content [7, 14, 19, 20]. In Malawi there is a guide compiled by the Ministry of Health which has specific topics for each antenatal care visit and some topics are repeated during subsequent visits [28]. Topics include: mother to child HIV prevention, birth preparedness and complication readiness plan (BP/CR), nutrition during pregnancy and male involvement. Despite the guide, other researchers have argued that the content of antenatal education does not take into consideration the needs of women in Malawi [16]. In an attempt to fill this gap, there is one study which assessed antenatal information needs of first time mothers. However, there is minimal information on such needs among men and women (couples), in Malawi hence the need for this study. Therefore, this research aims at exploring antenatal information needs for couples from different stakeholders to assist in the designing of a couple-centered antenatal education package. The package will be used by midwives to educate and inform groups of expectant couples during their routine antenatal care visits as an intervention which will be tested for acceptability, feasibility and effectiveness in a future research project.

## Methodology

### Design

We conducted a formative exploratory cross sectional descriptive study using a qualitative approach from February to August 2016. The design enabled the researchers to have a deep understanding of information that couples want during antenatal education [29]. We employed focus group discussions (FGDs), in-depth interviews (IDIs) and key informant interviews (KIIs). Focus group discussions allowed the research team to generate contextualized multiple viewpoints on information needs of couples during antenatal education [30]. In depth interviews provided detailed information on desired topics for couple antenatal education whereas key informant interviews allowed the generation of rich information based on the participant’s expertise [29, 31]. We conducted the following interviews; four focus group discussions among men and women independently plus an extra focus group composed of nurses/ midwives, 13 key informant interviews, 10 in-depth interviews with couples and 10 separate in-depth interviews with men who had been to antenatal clinics before with their spouses. The sample sizes for FGDs, KIIs and IDIs were guided by literature on reaching theoretical saturation [32–34]. Furthermore, the sample sizes allowed the researchers to recruit participants for FGDs, IDI and KII based on key demographic variables that were likely to have an impact on participants’ view on content for couple antenatal education [29, 33].

We followed RATS guidelines in presenting the manuscript including the results of the study (see Additional file 1: RATS Checklist).

### Study setting

The study was conducted in Blantyre District situated in the Southern region of Malawi in the following sites: Mpemba, South Lunzu, Queen Elizabeth Central Hospital, Blantyre District Health Office and Kamuzu College of Nursing.

### Health centres

Mpemba and South Lunzu (SL) health centres and their catchment areas are located in the southern and north-eastern parts of Blantyre District respectively. The District is divided into rural and urban settings. The urban setting is regarded as the main industrial city of Malawi as it contains industries and companies which provide employment. Mpemba and SL health centres serve both rural and urban communities. About 100 to 120 new pregnant women report for antenatal care at SL while Mpemba registers 80 to 100 per month. In both facilities antenatal services are provided free of charge. Additionally, antenatal education is an integral part of antenatal care and topics which are frequently discussed during antenatal education include birth preparedness and complication readiness planning focusing on items to prepare and prevention of mother to child HIV transmission (PMTCT). The topic of nutrition for a pregnant woman was discussed sporadically during antenatal education. Few men accompany their wives for antenatal services in both facilities according to anecdotal reports by maternity unit nurse/midwife in-charge at the facilities. Mpemba and SL health centres and their catchment areas were chosen because the centers are semi-urban and the views on content for couple antenatal education participants shared during interviews were likely to represent those of rural and urban populations of Blantyre District.

### Queen Elizabeth central hospital

Queen Elizabeth Central Hospital (QECH) is the largest public tertiary hospital in Malawi and functions as the main referral hospital for the Southern Region. It is also a teaching hospital for different health related professions. On average about 100 women report to QECH for antenatal care per month (Personal communication by nurse/midwife in charge). The facility offers antenatal care on paying basis. Few men escort their spouses for antenatal care at the facility. During antenatal education, different topics are discussed with much emphasis on prevention of mother to child HIV transmission, birth preparedness and developing a complication readiness plan, family planning, exercises and nutrition during pregnancy. The hospital was chosen to provide adequate professional nurses/midwives for FGDs, as SL and Mpemba health centres could not.

### Blantyre District health office and Kamuzu college of nursing

Blantyre District Health Office is responsible for managing health care services in all the health facilities situated in Blantyre. Kamuzu College of Nursing (KCN) is one of the institutions in the country which trains professional nurses/midwives and has lecturers experienced in midwifery education and practice. These institutions have individuals with different expertise in maternal health, including policy making, and can provide diverse opinions on preferred topics to be covered during antenatal education.

### Rangers security service company and plant and vehicle hire engineering services

Rangers Security Service Company (RSSC) and Plant and Vehicle Hire Engineering Services (PVHES) are non-healthcare settings which were used in this study. The former is privately owned institution while the latter is government owned. Most of the employees are unskilled male labourers whose partners access maternity health services from the public health facilities of Blantyre District. The sites were chosen because they had participants with different backgrounds, which broadened the understanding on antenatal education information needs for couples.

### Selection and recruitment of female participants for FGDs

Convenient sampling was used to recruit women attending antenatal clinics at SL and Mpemba health centres and the total number of the participants was 34. The principal investigator and a female research assistant approached the women in the waiting areas of antenatal and under five clinics. The women were informed of the study and those willing to participate were requested to remain after the services for informed consent procedures and discussions. Recruiting participants through the clinics provided an opportunity to observe antenatal education sessions and the topics taught to women. Women were divided into two age categories; the first category being those aged 18 to 25 years while the second category being aged 26 years and above. In total, there were 17 women less than 26 years of age and 17 greater than 26 years from Mpemba and South Lunzu.

The inclusion criteria for women to participate were: willingness to participate in the focus group discussions; expectant or had a child under five years during the period of the study; and were from the catchment areas of Mpemba or South Lunzu. Recruiting women with a child under five years meant the participants would be able to remember the childbirth experience. The women also varied in parity to determine similarities and variation on the subject matter. The discussions were conducted in a room at the health facilities. Four focus group discussions were conducted in total which were divided by the two age categories at each health facility.

### Selection and recruitment of male participants for FGDs

Purposive sampling was used to recruit men for FGDs with the help of health workers. Purposive sampling provided an opportunity to choose participants who had the knowledge and experience on the subject matter as they were likely to contribute to the study purpose [29, 35].

Variations such as place of identification, number of children, education and age were considered during the recruitment process. The men were classified into two groups: young men between 18 and 25 years and older men above 25 years. The first category comprised 16 participants (six from SL and 10 from Mpemba) and the second category had 19 participants (10 from SL and nine from Mpemba). The inclusion criteria for men were: willing to participate in the focus group discussions, able to give in consent, having an expectant spouse, having a child under five years during the period of the study, resident of the catchment areas of Mpemba or South Lunzu; and were above 18 years old. Just like women, the men were included in this study because they would be beneficiaries of the forthcoming intervention. All the participants who were approached for the FGDs accepted. However, two male discussants, one from SL and one from Mpemba did not appear for the discussions and did not give any reason for their absence. In total, there were four FGDs conducted, one for men ≤25 years of age and another for men > 25 years at each site. We agreed with the male participants on the appropriate date, time and venue for the discussions.

### Selection and recruitment of nurses/midwives for FGDs

Nurses/midwives were purposively recruited based on their roles and responsibilities in maternity care. Variations in terms of cadre, years of service and gender were considered in order to have a broader perspective of the content for couple antenatal education. Seven nurses/midwives were selected; two each from Mpemba and SL health centres and; the remaining three were from QECH as Mpemba and SL health centres could not provide the recommended number for a FGD. Three were registered nurses/midwives while four were nurse/midwife technicians and their experience ranged from four to 18 years working in maternity units including antenatal clinics. All were females except one. Nurses/midwives were included as we felt that through their training and experience they would be in a position to propose education content for couples. Although the group was heterogeneous in the sense that it had nurses/midwives from a central hospital whose exposure might be different from nurses/midwives from health centres thereby affecting discussions, the advantage was that data were enriched as it came from different locations from people with different experiences.

### Selection and recruitment of key informants

Key informants (KIs) were selected based on their roles and responsibilities in various institutions, which were both health related and non-health related, to gather their perspectives in relation to messages couples should receive when they come for antenatal education sessions. The researchers recruited the key informants. In total, there were 13 KIs and the composition was as follows: one obstetrician, one senior nurse/midwife educator (Head of Department for Maternal and Child health), one senior practicing nurse/midwife (matron), one policy maker (maternal and child health coordinator). Those from non-health institutions were two religious leaders, one Christian and one Muslim, one leader representing the small-scale business community, two group village heads (female and male) and four employers (two from private owned institutions, one from a statutory cooperation and one from a public institution).

The KIs with health backgrounds were chosen as they would provide relevant content for couple antenatal education. Similarly, we assumed that non-health KIs, due to their diverse backgrounds, roles and responsibilities, that their input would not be biased in relation to content for couple antenatal education. For instance, we chose two religious leaders, one Muslim and the other Christian. They were chosen because they represent the two major religious groups in Malawi and would provide a religious point of view. Additionally, the village heads (one male and one female) would suggest topics for couple antenatal education which are likely to be culturally acceptable.. The eligibility criteria for KIs required them to be 18 years or above and currently serving in that particular position for not less than two years. All key informant interviews were conducted at the work place during the convenient time and date agreed upon. The interviews were conducted in a private place.

### Selection and recruitment of couples and men who had previously escorted their spouses for antenatal care for IDIs

Participants for IDIs were men who had escorted their wives to the antenatal clinic previously, three years prior the study and couples who were expectant or had a child under five years during the period of the study and were from the catchment areas of Mpemba or South Lunzu. Men who had escorted their wives were chosen in order to ascertain their opinions as they were already motivated to be involved in antenatal care and would therefore be in a better position to share relevant antenatal education information couples may need. Couples were chosen because this study sought information for couples, therefore their input as couples could be of great value. Both the men and couples were aged 18 years and above. With the assistance of health workers, the participants were recruited through the health facilities and most of them were recruited from the catchment areas, as with the male discussants. All the participants who were approached for the IDIs consented to the interviews. Interviews were conducted at agreed times and venues. The participants varied in terms of age and number of children. In total there were 10 IDIs for couples and 10 men. Observing variations during recruitment allowed us to identify differences and similarities among the groups of participants in relation to the subject matter, which in this study was the content for couple antenatal education.

### Data collection for FGDs

Two research assistants (RAs), a female registered nurse/midwife and a male social scientist, were recruited. The RAs were recruited as they have knowledge on maternal health, male involvement and the research process. In addition, the presence of a male research assistant might have helped male participants to openly share during FGDs for men. The research assistants received training to familiarize themselves with the study, its background, aim and their roles and responsibilities in this process of data collection.

The participants were given information regarding the purpose of the study and why they were chosen including the time each interview would take which was 60 to 90 min. To facilitate their understanding about the study, the participants were given an information sheet and were asked to read it carefully. For those who could not read, the information sheet was read to them. They were informed that they could ask questions for clarification and that their participation was voluntary.

Additionally, it was communicated to the participants that the discussions will be digitally recorded for accuracy and completeness of data, which all participants accepted. All participants signed an informed consent form demonstrating their acceptance to participate in the study.

All focus group discussions except the male FGDs were moderated by one of the researchers (MCC) while the research assistants filed verbal and nonverbal behaviors of participants during discussions and recorded the conversations. The male FGDs were moderated by the male research assistant and MCC was the note taker. The participants in the FGDs were given numbers for identification during the discussions. All FGDs, except with nurses/midwives, were conducted in the local language, Chichewa. The FGD for nurses/midwives was conducted in English, the language of instruction in schools and work places. A pretested unstructured discussion guide was used and had one broad question as follows: What type of information should be discussed with expectant couples during antenatal education sessions? Probes were used during the discussions to obtain detailed and clear information about antenatal education information needs for the couples. For example, participants were asked to suggest the content and depth of discussion concerning pregnancy, childbirth and the postpartum period (see Additional file 2: Focus group discussion guide). The unstructured discussion guide generated rich information as prior information on the subject matter did not influence the interview, rather the participants’ narrations structured the interview [29, 36]. After each interview, a summary of important points regarding antenatal information needs was provided to the participant as means for verifying what had been discussed during the discussions [29]. Additionally, one of the researchers (MCC) and the moderator met to review and plan for the next focus group discussion after each FGD.

### Data collection for IDIs and KIIs

One of the researchers (MCC) conducted all the in depth and key informant interviews using the pretested unstructured interview guides which had one broad question and probes just like the discussion guide (see Additional file 3: In depth interview guide and Additional file 4: Key informant interview guide). Each interview took 40 to 90 min and none of the participants refused to have their voices recorded. All IDIs and some KIIs were conducted in Chichewa. After each interview, a summary of important points regarding antenatal information needs was provided to each participant as means for verifying what had been discussed during the interviews [29].

### Trustworthiness of the data

To ensure trustworthiness of the data, we considered credibility, dependability, conformability and transferability. Piloting of the research instruments and inclusion of direct quotes in the results section enhanced credibility and dependability. We achieved conformability by triangulating data as information collected from multiple sources and methods help in confirming emerging issues [29, 36]. The study considered parity of the participants, which enhanced transferability of the study as views came from individuals with a variety of experiences. In addition, the participants were drawn from semi-urban settings, which would mean we captured views from urban and rural settings.

### Data management

The recorded information, field notes and transcripts were kept in a lockable cupboard accessible by the researchers only. The computer with the data had a password known by one of the researchers (MCC). Soon after data collection, the researchers transcribed the recorded data verbatim and the Chichewa transcripts were then translated into English. Recorded data were transcribed verbatim before cleaning and anonymising to remove any participant identifying details.

### Data analysis

Data analysis was done simultaneously with data collection to allow the researchers refine the subsequent interviews. The data were analyzed using thematic content analysis frame work. Braun & Clarke [37:79] describe thematic analysis as “a method for identifying, analyzing and reporting patterns (themes) within data not necessarily on dependent on quantifiable measures but rather on whether it captures something important in relation to the research question [37]”. Conversely, content analysis, apart from identifying, analyzing and reporting themes, patterns of words used and their frequency are also regarded important as frequent occurrence may indicate significance particularly in areas where little is known about a particular phenomenon [29, 35]. In this study, thematic content analysis was significant as it involved exploring views from different stakeholders on the content for couple antenatal education, an area with little information. Deductive and inductive approaches were employed to code the data. The former focused on literature and study objectives while the latter was data driven. The data analysis was guided by six stages according to Braun & Clarke which are familiarizing with the data, generating initial codes, searching for themes, refining themes, naming themes and producing the report [37].

### Familiarization with the data

All the scripts for the focus group discussions, in-depth interviews and key informant interviews were read once by MCC against the recorded information to get the sense of the data. Initial ideas related to the objective of the study were written down. Thereafter, identification numbers for each script were written on small pieces of paper and casted in a box. An independent person picked four papers from the box as the researcher wanted four transcripts for further familiarization with the data. The four chosen transcripts were as follows: couple in depth interview no 1 (CID 01), older male focus group discussion from South Lunzu, key informant from PVHES (Key informant No 11) and a younger female focus group discussion from Mpemba.

### Generating initial codes

We coded a transcript (couple IDI CID 01) by reading through line by line. All items relating to the same topic were coded to similar nodes. Coding was done inductively and deductively.

### Searching for themes

Codes that were identified in stage two above were reviewed by an independent researcher who also coded a clean copy of the script which MCC coded. Agreement was done on the codes to be used for all the transcripts with the independent researcher, co-authors: ASM and EMC. The codes identified were used to code the data and emerging codes were included as well in the process. The coded data was categorized and common themes were identified.

### Reviewing themes & defining and naming themes

We reread the data to identify a coherent pattern and to see if the data fitted into each theme identified. The first author verified the themes by checking on them against the audio taped data. Similarities and differences were noted across the data set at this stage.

### Producing the report

The report outlined the overarching theme, subthemes and categories.

### Ethical approval

We obtained ethics approval from the College of Medicine Research and Ethics Committee (COMREC) Certificate No P.11/151821. Permission was obtained from individual heads of institution where participants were drawn as follows: Queen Elizabeth Central Hospital (QECH) Blantyre District Health Office, Blantyre District Commissioner, Kamuzu College of Nursing, Blantyre Campus, Private Vehicle Hire Engineering Services (PVHES) and Rangers Security Company. Additionally the researchers ensured the participants were aware they could withdraw without reprimands at any time. Confidentiality and anonymity of the participants were observed by conducting interviews in a private room and using codes for identification of the participants. Participants in focus group discussions were told that the researchers were not in control of the information that may be disclosed outside of the discussion by participants within the group itself. The participants were also informed that the information collected might be published while maintaining confidentiality as agreed.

## Results

### Demographic characteristics

Out of the 119 participants for this qualitative study, 66 were men and 53 were women. Thirty- four women and 35 men participated in the focus group discussions. Focus group participants were the youngest with median age 26.0 (inter quartile range [IQR]: 24, 32). Key informants were the oldest with median age of 46.0 (IQR: 40, 58). All the participants were married except 4 from the category of focus group discussants. The number of years spent in marriage was lowest among focus group discussants with median of 4 years in marriage (IQR: 2, 8) and highest among key informants with median 17 years in marriage (IQR: 10, 28). All the participants had attended formal education except 5 who were one from Key informant group and 4 from focus group who had no education. The majority of the participants 50 (42.1%) had reached secondary education followed by primary education 36 (30.2%) of the participants. Few participants reached tertiary education 28 (23.5%) and 5 (4.2%) had no education. The key informants had the highest number of children median 3 (IQR: 2, 5) with the lowest number of children among focus group discussants median 2 (IQR: 1, 3).

### Theme identified

We identified one overarching theme: information for couple antenatal education service. Following were three subthemes which were aligned to the three domains of the maternity cycle (antepartum, intrapartum and delivery and postpartum period). Each subtheme had several categories which are presented as topics (see Additional file 5: Distribution of subthemes).

#### Antepartum education needs

A variety of topics were mentioned as follows with some variations among the groups.

##### Description of pregnancy

A few participants felt that couples should be taught about pregnancy including the changes which occur when one is pregnant. Furthermore, the participants had the view that with education, men will become knowledgeable and understanding about pregnancy issues. This would lead to men providing the needed support to their spouses as summarized in the quotes below:

*“They need also to know what happens to the woman when she’s pregnant psychologically. This woman she should be okay because if they don’t know the physiology of pregnancy, the process of pregnancy, it will be difficult for the men to support their wives”. Key Informant No 6 (KI No 6).*

*“I would love to know what happens during pregnancy … .so I want that topic that when a woman gets pregnant….”. Focus group male (FGM) Mpemba p4.*

##### Care of a pregnant woman

Most of the participants mentioned care of a pregnant woman as an important factor. The participants expressed that caring for a pregnant woman as a topic was core because it would yield a healthy baby. They went into detail with nutrition and a few participants mentioned rest and emotional care as depicted in the quotes below:

*“First we need to have information on the nutritional requirements for the pregnant woman because a healthy baby will come from a mother who is well fed”. Couple (Husband) p 10 “I heard that they [pregnant women] require rest and should not do hard work, they should be made happy … so that after nine months we have a better outcome”. FGM, South Lunzu (SL) p5.*

*“Men should be taught how they can take care of pregnant women”. FGF, Mpemba p3.*

##### Role of men during perinatal period

The role of men during the perinatal period was mentioned by male participants and a key informant with a health background as one of the preferred topics for expectant couples as per quotes below:

**“** *Maybe if we could also learn that, the responsibility men have on the baby, even after it’s born”. Male in depth Interview participant No 9 (MII p 9).*

*“I think they (teachers of antenatal education) should emphasize on the role of men during pregnancy and delivery when they are teaching because most of the times the nurses ask them who is your helper during this period and they respond my mother, which means even the health personnel they feel that it’s the role of women not men to care for a pregnant woman.”. FGM, SL p6.*

*“When you are giving the health education, all the topics when you are giving there should be information about how men can be involved in each and every topic. Let’s say nutrition, why is it important for men to be involved in nutrition …” KI No 6.*

None of the women mentioned the role of men as a preferred topic. However, one woman from the younger group mentioned the topic of respect and care for men. She felt that the antenatal education should also include giving attention to men instead of only the delivered baby during the postpartum period in order for the men to feel included and had this to say:

*“Maybe the way how we can respect them. A theme that if they hear it, they can be pleased ….maybe because we have a kid, we should be caring only the kid, no we should also take care of him”. Focus group female (FGF) SL p7.*

##### Family life

Key informants from non-health institutions and men who have been to antenatal clinics with their spouses felt that couples should be taught on family life which should cover parenting and relationships within a family as reflected in the quotes below:

*“There should be that topic of encouraging men to change their association with the family … To improve their association with the family … this is very important”. KI No 1.*

*“Topics should concern how to live in families, man should not abuse his wife and* vice versa *… You may teach about domestic violence in the households. Men are abusing women and women are abusing men in the homes”. MII p 6.*

##### Birth preparedness and complication readiness plan (BP/CR)

Younger male and female participants; nurses/midwives; key informants like chiefs; men who had escorted their wives to antenatal clinics before; and couples, especially the wives, mentioned birth preparedness as a preferred topic. Participants reported that men should have information on birth preparedness as it would enable them to participate and meet the requirements as heads of families. The components of birth preparedness included items to prepare for delivery with a few mentioning danger signs, funds and the need for a companion as shown in the quotes below:

*“I can be happy if we are to be taught how to prepare for childbirth, like the clothes, buckets, anything that is needed at the hospital, even the baby’s clothing, it would be better to teach us those”. FGF SL p2.*

*“I think when we come together you can tell us about the dangers associated with pregnancy for example the swelling of feet, fainting, loss of blood, birth preparedness here and there, these things we can learn”. FGM Mpemba p4.*

##### Prevention of mother to child HIV transmission and syphilis testing

Some participants mentioned prevention of mother to child HIV transmission (PMTCT) as an important topic. They were discussants from nurses/midwives; young male groups; a key informant; a man who had escorted his wife to the clinic before and couples; especially husbands. The quotes below summarize what was discussed:

*“The first thing I would wish men and women to learn together and should happen while they are together is HIV testing….” Focus group nurses/midwives participant No 6 (FGN/M P6).*

*“What I want most is factors which can put the mother and baby at risk. In fact, we are supposed to know our status before becoming pregnant so that we can protect the baby many men do not know things which can put the baby or mother at risk so they need to teach us about these things”. FGM SL p5.*

##### Sexual activity during pregnancy

Older male discussants, nurses/midwives, couples (husbands only), a key informant and a man who had escorted his wife before to the clinic mentioned sex during pregnancy as the preferred topic in relation to pregnancy. Participants were in a dilemma on which information to follow regarding sex during pregnancy. Informally, they were taught to stop at different gestational ages of pregnancy depending on one’s culture. None of the female and young male participants in the study mentioned sexual intercourse during pregnancy as preferred topic.

*“I wish to know, because the elders tell us that the limit is seven months, to stop having sex with a wife. What if you proceed up to the ninth month, or even when your wife will give birth tomorrow and you have sex today, what would happen”? FGM Mpemba p7.*

*“I think men should be taught mostly on how they can be with their wives; a lot of men sleep with their wives yet they are given months to say these months you should not sleep with your wife but so many men don’t want to leave their wives so if they are told to do so,”. Couple (Husband p8).*

*“I think one topic they would want to know and have interest with it would be on intercourse, the period they could have and may not have sex, because most are not aware of this, they are told from their homes to stop when she’s seven months pregnant and to resume six months post-delivery”. FGN/M p4.*

Older male and nurses/midwives mentioned sexual position as one of the topics to be included. Pregnancy becomes a barrier to sexual activity and there was a need for couples to learn the positions so that conjugal obligations are maintained, as per expressions below:

*“So if the hospital can be inviting us to hear those things [sexual positions] … because sometimes we are carried away you may find yourself doing it on top at eight or nine months gestation which may not be acceptable (participants 2, 5 and 7 agree in the background)”. FGM old SL p9.*

*“We should … include the positions, sexual positions when the woman is pregnant because sometimes the woman still has the feeling but then what is making them to fail is maybe the fundus (pregnancy) is big that time so I think we should also educate them on the sexual positions that they can use that can be comfortable to the pregnant (woman)” FGN/M p3.*

Although culture was questioned in relation to sex during pregnancy, older female discussants and a key informant felt that some informal teachings, which are emphasized traditionally, should be taken on board during couple antenatal education. One of these was prohibiting husbands’ extramarital affairs when the wife is pregnant in order to promote their health as in the quote below:

*“We can be happy if the hospital can also take the cultural aspect by advising the men not to have extramarital affairs when the wife is pregnant and the effect of such behaviors should be emphasized during the couple antenatal education. Maybe this can help in the sense that men will be afraid and can take care of themselves”. FGF SL p3.*

#### Intrapartum education needs

Participants were asked to explain areas which should be discussed during couple antenatal education concerning child birth and the subtheme had two categories as follows:

##### Signs of labour

Signs of labour were mentioned by nurses/midwives, female young focus group discussion, few couples, men who had escorted their wives to antenatal clinic before and some key informants as a topic to be given to couples as it would facilitate spouses to have timely delivery by skilled attendants as captured in the quote below:

*“The other thing they are supposed to know is the signs of labour and delivery … if they see these signs they are supposed to go to the hospital quickly”. FGN/M p1.*

*“Everything like signs of labour … so these are the kind of issues that men need to hear and will not have problems with such teachings”. FGF SL p1.*

*“….When we are learning like you can emphasize on the topic of labour …. ….if she comes to hear it like here at the hospital, when you give us your messages …the woman is able to leave home early, reach the hospital (for delivery) without giving any problems”. KI No 2.*

##### Giving birth

Delivery and its possible outcomes were mentioned by the majority of the participants as preferred topics. Participants were of the view that since men culturally are not allowed to observe their spouse giving birth and that delivery suites particularly in public health facilities cannot accommodate male partners, they felt men should be taught with their spouses on this topic. They felt when men have the knowledge on labour and delivery, they will understand and appreciate what women go through and can assist accordingly.

*“Mostly we would like to know issues about giving birth, since women say that we shouldn’t beat the child because they felt pain to give birth to that child (laughter) so we would wish to know that what exactly happen”. FGM Mpemba p3.*

*“I would also like to know when labour has started … Sometimes labour can start when the elders are away or busy … so that would mean going to those places to call them. In the meantime your friend is struggling instead of you assisting her on the way to the hospital or if there are complications on the way to the hospital if those women are there you should work together when they say bring that you should also be participating because in so doing you can save the life of the mother as well as the baby”. FGM Mpemba p 4.*

*“When the men come, we should teach … the process of labour….., teaching them everything up to the point when he baby is born. …to make them have a picture on what may happen and what may not happen”. FGN/M p3.*

#### Postpartum education needs

Education content for postpartum period had categories such as sex after delivery, baby care, danger signs of a baby and family planning.

##### Sex after delivery

Older male discussants from Mpemba and nurses/midwives, a few couples, key informants and men who had escorted their wives before to the clinic proposed sex after delivery as a topic for couple antenatal education. Participants expressed that this topic was crucial for couples because what they learnt from the hospital was different from what they had learnt from home (from the elders). They sought clarity on this matter as per quotes below:

*“Because the elders tell us that when the baby is born, you should wait for six months before having sex, they say that all the bad things should end. So should we follow the elders’ rules? That we wait for 6 months or not?*” *. FGM Mpemba p2.*

*“When the baby is born some are told there that you should just stay for 2 weeks since nowadays men are more lustful so wouldn’t you find any problem? I would like to know about that”. FGM Mpemba p9.*

*“It is also important to make a man aware that when a woman has delivered, she has a given period when her organs should go back [pre-pregnant state] (laughter). I am saying this because some men don’t understand and they are unable to hold themselves, you find that within two weeks (other respondents in the background comment it is within days) the man has already started bugging for sex …. Because we just say six weeks, we should let them know that when a woman has …delivered, the woman can be in pain that would make her not to feel the pleasure of sex (laughter) yes because when you tell them that they will fear episiotomy otherwise they cannot fear it”. FGNM p7.*

##### Baby care

Baby care, which focused on feeding the baby was mentioned by some participants as a priority topic for couple antenatal education as expressed in the quotes below:

*“They can also talk about how to take care of the baby after delivery so that she grows in good health”. FGF Mpemba p8.*

*“Men are also supposed to know about exclusive breast feeding and breast attachments ….so if the men know this they would give their wives time to rest”. FGN/M p5.*

*“Postnatally how to take care of the gift [baby]. We need to learn all these things together”. FGM SL p6.*

##### Danger signs of a baby

One participant felt that couples should also be knowledgeable about danger signs a baby might experience as per the quote below:

*“What problems can our babies’ experience? All those we need to teach them (couples)”. Couple (Husband p1).*

##### Family planning

Family planning was mentioned by the majority of participants. The participants felt that this information was important for couples as it would aid compliance to family planning methods as per quotes below:

*“If I come with my husband, I would like to receive education on family planning, so that ….husband should participate in family planning”. FGF Mpemba p1.*

There was a general feeling among some participants that topics should be decided by hospital personnel as they are aware of the needs. Additionally, key informants with health backgrounds felt that the topics which are given to women can be given to men as well when they accompany their spouses to the antenatal clinic as reflected in the quotes below:

*“We are like learners we cannot choose what to learn. As a teacher you know what learners need because you went to college”. FGM SL p9.*

*“The health care workers should teach us what they know, because we just stay in the village, we know nothing” (other participants agreeing)*. F*GF Mpemba p7.*

*“I don’t think there are any topics that we teach …that I would say no …..I think what this woman should know is the same what this man and woman (Couple) should know”. Key informant p3.*

## Discussion

We found that antenatal information needs were relatively similar among men and women. On one hand, care of a pregnant woman, giving birth, baby care and family planning were the preferred topics for both men and women in this study. On the other hand, sex and men’s roles during the perinatal periods, PMTCT and family life were the desired topics mentioned by male participants as compared to birth preparedness which was mentioned more by the female discussants.

In this study, a description of pregnancy was one of the preferred topics for couples during antenatal education sessions as reported by other studies [16, 18, 21]. This could be the case because pregnancy is associated with physical and psychological changes which women and their families need to cope with, but are only able when they have adequate knowledge on what pregnancy is [21]. Additionally, the topic is rarely taught during antenatal education sessions in Malawi, hence the need for this topic. Congruent to previous studies [15–17], our study shows that care of the pregnant woman was a preferred topic by both men and women. This is probably the case because men and women were aware that care can influence pregnancy outcomes, therefore the need for more information among couples to support each other. However, participants did not mention emotional support for men as a preferred topic as reported in other studies from western settings [11, 21, 38]. The varying cultural backgrounds in the different settings and the socially accepted sense of masculinity may explain the non-recognition of male emotional support because such a man may be viewed as weak. Another topic which was not reported in this study is on traditional beliefs and taboos related to childbirth which was reported by women in another study in Malawi [39]. Although the participants did not mention the topic as a preference, we strongly feel that harmful cultural beliefs can negatively influence the outcome of pregnancy. Therefore, we suggest that couples need to learn the topic and should be included in all the three domains (antenatal, labour and post-delivery) since the beliefs can be practiced at different stages of the maternity cycle.

Birth preparedness and complication readiness plan (BP/CR) focusing on items to purchase in preparation for childbirth were mentioned as a preferred topic in this study by a majority of the participants with a few suggesting the inclusion of danger signs during pregnancy. This is in agreement with other studies [16, 40–42].

Including danger signs in birth preparedness and complication readiness plans during couple antenatal education is significant as it is likely to improve male partners’ involvement in meeting the demands of BP/CR, as reported by Weldearegay and others in Ethiopia [43, 44]. In this study, the fact that women mentioned items to purchase as part of BP/CR demonstrates that the current BP/CR strategy is not adequate. This may affect the outcome of the strategy, which is to prevent unnecessary delays in accessing emergency obstetric care and connecting women and their families to skilled birth attendants [45, 46]. We recommend that BP/CR information be complete in order to achieve its intended purpose. The information should include: recognizing danger signs and making proper arrangements to seek skilled care; identifying a health facility for delivery; identifying and saving funds for transportation; and purchasing materials like clothes for the mother and baby for delivery.

In this study, men recommended the topic of their own roles during the perinatal period. Other studies [19, 47, 48] have echoed these findings by suggesting that men can be partners in maternal health if their roles are known. This will assist in reducing men’s stress as they assimilate into the domain of parenting. As stated by [38] and Brown, participants felt frustrated and excluded when ignorant of their roles in maternal health care; this prevented them from participating [48]. Adolescents wanting to learn their roles as parents made similar observations as they recognized that they had no preparation for these responsibilities [49]. Opondo [50] argues that it is not only the father’s physical activities which would benefit a baby, but the father’s emotional state can influence the outcome as well. We feel that fathers’ preparation for their roles should go beyond the knowledge and skills and focus on the emotional aspect if male involvement is to be improved. This could be achieved by emphasizing the benefits of male involvement during antenatal education to couples.

Some participants preferred PMTCT as an important topic. This is probably because Malawi, among other countries, has low rates of male involvement in PMTCT programmes [51–53]. Therefore, through the antenatal education sessions men may have information which may drive them to participate in PMTCT services; PMTCT is one of the services offered within antenatal health services.

Family life was another suggested topic to include during antenatal education, as reported by Axelsen [20]. This is against a background where the perinatal period may be a source of marital strife [54–56]. Stressors include lack of sexual relations, unfamiliar baby care and adapting to parenting. Therefore, positive familial relationships are core to male involvement in spheres labelled as female domains, as reported by Nyondo et al. and Larsson et al. [57, 58]. In our study, gender based violence and respect for male partners were unique topics for couples, as they would facilitate male partner involvement in maternal health, ultimately improving maternal outcomes [59].

Labour remains crucial in couple education as documented by other studies [16, 17, 60, 61]. In this study, participants wanted men to learn about labour and delivery issues, which was similar to a study [62] which found that men felt more satisfied with these issues as compared to parenting. Participants wanted content on labour and delivery as it would assist male partners intervene in a timely manner. Normally in Malawi, a pregnant woman would be assisted by fellow women within the neighborhood, but due to economic pressure, growing individualism and the dilution of social cohesion this is no longer the case. Hence the need for male partners to become knowledgeable on maternal health issues. Although culturally men are not accepted to observe a spouse giving birth as expressed by some participants in this study, the health sector allows men to observe and assist their spouses in the delivery suite. However, the present infrastructure of the delivery suites/rooms in most of the public health facilities cannot accommodate male partners. In some private health facilities whose delivery suites accommodate men, few of the men accept to observe their partners delivering [63].

Consistent with previous studies [14, 26], family life which reflects parenting and relationships within a family is another topic which was chosen for couples’ education. This may be because of a shift whereby men want to be responsible fathers and engaged in the upbringing of their children [9, 11]. In addition, studies have shown that some men are unprepared for parenthood therefore antenatal classes would provide a solution to this gap [14].

Sex during and after pregnancy was the other topic suggested by male participants. Women were mostly too shy to talk about sex, as echoed by Pauleta and Naim [54, 64]. Studies from high income countries have reported on sex before and after birth as preferred topics too. The difference with previous studies is that, in this study, men were more concerned with the cultural norms when one is pregnant. In previous studies, the primary concern was about sexual positions and consideration for the growing fundus and tiredness during pregnancy. In some communities in Malawi and elsewhere, sex before delivery is associated with the belief that it will make the baby ‘dirty with sperms’ and injured. While sex after delivery is associated with the idea of contamination from the lochia, which can harm the man and the baby [54, 55]. Therefore, men were keen to know about these topics because they are likely to be the ones to initiate sex [56]. Additionally, the traditional teachings contradict modern information promoted in hospitals regarding coitus during pregnancy and post-delivery. Therefore, men wanted verification from a reliable source. This is in line with what was reported by Williamson [65]. Health personnel do not give adequate information on sex, such as when to resume sex, yet they are seen as a reliable source for health information. In this study, even the nurses/midwives were not sure as to when couples should resume sexual intercourse after delivery. Some mentioned two weeks while others mentioned six weeks post-delivery. There is a need to clarify and provide reasons for the fact that sexual intercourse should resume six weeks after delivery, as stipulated in the sexual health policy of Malawi [66]. However, it is worthy clarifying that six weeks is just a guide as some may not feel comfortable to resume sex at that period due to slow and poor recovery following childbirth. Therefore, the emphasis during the teaching should be that sex can be resumed after 6 weeks post-delivery when the woman has fully recovered from child birth.

Baby care was another preferred topic which is congruent to the needs reported by men and women from other studies [4, 19].

However, in this study participants were concerned with gaining knowledge on maternal and neonatal care as opposed to studies from high income countries which indicated men were interested in infant psychomotor skills [48, 50, 67]. In the Middle East, men and women felt that baby care activities such as bathing and changing nappies should not be learnt by men [68]. Although disparities exist, our study suggests that the current antenatal education, which focuses on theory, should consider including demonstrations of skills associated with maternal and neonatal health in order to motivate men. This is in a context where men are likely to feel in control when they know a practical tangible skill [27]. Further men, as adult learners, have life experiences and prior knowledge and can benefit from active involvement through the learning of skills.

Participants also mentioned the topic of family planning as a preference for couples’ education, which was also mentioned a decade ago in Malawi [16]. The reason could be there is a greater preference for small families, due to social and economic pressures. Moreover, men are blamed for slow progress in family planning programmes [69, 70]. Therefore, knowledge about family planning may facilitate men’s participation.

There was a general feeling among participants that the hospital is the steward of topics and can decide what couples can learn. This is contrary to what is being suggested by researchers, that both men and women have their own learning needs, which need to be addressed. In support of this view, Ho found that women attending antenatal classes in China expressed that the learning was not organized around their life situations. Rather, it was according to the subject matter, and as a result the women found it difficult to remember [3]. Similarly, participants in Anderson’s study expressed the need for midwives to focus less on medical issues and concentrate more on parents’ and partners’ perspectives of childbirth [8]. In support of these views, Noronha asserted that Information education and communication material should be based on the scientific needs of the targeted audience [71]. Hence, content for antenatal education should be contextual and individual information needs should be taken into account.

### Strengths and limitations of the study

We consider the use of participants from different backgrounds, age and gender as a strength since information generated came from different perspectives. Additionally, triangulation of methods enriched the data further as the methods complemented each other. A possible weakness in this study could be participants giving responses that would please the facilitator, whom they knew had a medical background. However, this was minimized by techniques such as probing and member checking, which were employed to ensure that the responses were reliable.

## Conclusion

Antenatal education is a potential platform to inform and discuss with male partners about the childbearing period and early parenting. Therefore, if both men and women are to participate in antenatal education, their information needs must be prioritized. Men and women had similar choices of topics to be taught during couple antenatal education, with some minor variations.

## Additional files

Additional file1: RATS checklist. (DOCX 24 kb)

Additional file 2: A guide for focus group discussion. (DOCX 17 kb)

Additional file 3: A guide for in depth interviews. (DOCX 17 kb)

Additional file 4: A guide for key informant interviews. (DOCX 17 kb)

Additional file 5: Distribution of subthemes. (DOCX 18 kb)

## Abbreviations

BP/CR: Birth preparedness and complication readiness plan
CARTA: The Consortium for Advanced Research Training in Africa
COMREC: College of Medicine Research Ethics Committee
FGD: Focus group discussion
FGN/M: Focus group nurse/midwife
FGOF: Focus group old female
FGOM: Focus group old male
FGYF: Focus group young female
FGYM: Focus group young male
IDI: In depth interview
KCN: Kamuzu College of Nursing
KI: Key informant
KII: Key informant interview
PMTCT: Prevention of mother to child HIV transmission
PVHES: Plant and Vehicle Hire Engineering Services
QECH: Queen Elizabeth Central Hospital
RSSC: Rangers Security Service Company

## References

[1] Serçekuş P, Mete S (2010). Effects of antenatal education on maternal prenatal and postpartum adaptation. J Adv Nurs.

[2] Taiwo R, Salami F. Discourse acts in antenatal clinic literacy classroom in South-Western Nigeria. Linguistik online. 2013;31(2). https://bop.unibe.ch/linguistik-online/article/view/544/916

[3] Ho I, Holroyd E. Chinese women's perceptions of the effectiveness of antenatal education in the preparation for motherhood. J Adv Nurs. 2002;38. 10.1046/j.1365-2648.2002.02148. 10.1046/j.1365-2648.2002.02148.x 11895533

[4] Geçkil E, Şahin T, Ege E (2009). Traditional postpartum practices of women and infants and the factors influencing such practices in south eastern Turkey. Midwifery.

[5] Liu N, Mao L, Sun X, Liu L, Yao P, Chen B (2009). The effect of health and nutrition education intervention on women's postpartum beliefs and practices: a randomized controlled trial. BMC Public Health.

[6] White G. You cope by breaking down in private: fathers and PTSD following childbirth. Br J Midwifery 2007;15(1). DOI: 10.12968/bjom.2007.15.1.22679

[7] McElligott M (2001). Antenatal information wanted by first-time fathers. Br J Midwifery.

[8] Andersson E, Norman Å, Kanlinder C, Plantin L (2016). What do expectant fathers expect of antenatal care in Sweden? A cross-sectional study. Sex Reprod Healthc.

[9] Leverett S. Men and antenatal pedagogy: discourse, subject positions and affect: Cardiff University; 2013. URI: http://orca.cf.ac.uk/id/eprint/56523

[10] Tweheyo R, Konde-Lule J, Tumwesigye NM, Sekandi JN (2010). Male partner attendance of skilled antenatal care in peri-urban Gulu district, northern Uganda. BMC Pregnancy Childbirth.

[11] Smyth S, Spence D, Murray K. Does antenatal education prepare fathers for their role as birth partners and for parenthood? Br J Midwifery. 2015;23(5). 10.12968/bjom.2015.23.5.336.

[12] Singh D, Lample M, Earnest J (2014). The involvement of men in maternal health care: cross-sectional, pilot case studies from Maligita and Kibibi. Uganda Reprod Health.

[13] Yende N, Van Rie A, West NS, Bassett J, Schwartz SR. Acceptability and Preferences among Men and Women for Male Involvement in Antenatal Care. J Pregnancy. 2017;2017. DOI: 10.1155/2017/4758017. 10.1155/2017/4758017 PMC5294384 28243473

[14] May C, Fletcher R (2013). Preparing fathers for the transition to parenthood: recommendations for the content of antenatal education. Midwifery.

[15] Nigenda G, Langer A, Kuchaisit C, Romero M, Rojas G, Al-Osimy M (2003). Womens' opinions on antenatal care in developing countries: results of a study in Cuba, Thailand, Saudi Arabia and Argentina. BMC Public Health.

[16] Malata A, Chirwa E (2011). Childbirth information needs for first time Malawian mothers who attended antenatal clinics. Malawi Med J.

[17] Otaiby TA, Jradi H, Bawazir A. Antenatal education: Assessment of pregnant women knowledge and preferance in Saudi Arabia. J Women’s Health Care. 2:139. 10.4172/2167-0420.1000139.

[18] Singh D, Newburn M, Smith N, Wiggins M (2002). The information needs of first-time pregnant mothers. Br J Midwifery.

[19] Deave T, Johnson D, Ingram J (2008). Transition to parenthood: the needs of parents in pregnancy and early parenthood. BMC Pregnancy Childbirth..

[20] Axelsen SF, Brixval CS, Due P, Koushede V (2014). Integrating couple relationship education in antenatal education–a study of perceived relevance among expectant Danish parents. Sex Reprod Healthc..

[21] Widarsson M, Kerstis B, Sundquist K, Engström G, Sarkadi A (2012). Support needs of expectant mothers and fathers: a qualitative study. J Perinat Educ.

[22] Andersson E, Small R (2017). Fathers’ satisfaction with two different models of antenatal care in Sweden–findings from a quasi-experimental study. Midwifery.

[23] Andersson E, Christensson K, Hildingsson I (2012). Parents' experiences and perceptions of group-based antenatal care in four clinics in Sweden. Midwifery.

[24] Pålsson P, Persson EK, Ekelin M, Hallström IK, Kvist LJ (2017). First-time fathers experiences of their prenatal preparation in relation to challenges met in the early parenthood period: implications for early parenthood preparation. Midwifery.

[25] Adeniran AS, Aboyeji AP, Fawole AA, Balogun OR, Adesina KT, Adeniran PI (2015). Male Partner’s role during pregnancy, labour and delivery: expectations of pregnant women in Nigeria. Int J Health Sci.

[26] Svensson J, Barclay L, Cooke M (2008). Effective antenatal education: strategies recommended by expectant and new parents. J Perinat Educ.

[27] Premberg A, Lundgren I. Fathers' experiences of childbirth education. J Perinat Educ 2006. 2006;15(2):21–28. DOI: 10.1624/105812406X107780.

[28] Malawi Government Participants' manual for intergrated in service training for maternal and neonatal care, Malawi. 2014.

[29] Polit DF, Beck CT. Essentials of nursing research: appraising evidence for nursing practice: Lippincott Williams & Wilkins; 2013.

[30] Polit DF, Beck CT (2010). Essentials of nursing research: methods appraisal and utilization.

[31] Boyce C, Neale P (2006). Conducting in-depth interviews: a guide for designing and conducting in-depth interviews for evaluation input: pathfinder international Watertown, MA.

[32] Marshall B, Cardon P, Poddar A, Fontenot R (2013). Does sample size matter in qualitative research?: a review of qualitative interviews in IS research. J Comput Inf Syst.

[33] Morse JM. Critical analysis of strategies for determining rigor in qualitative inquiry. Qualitative health research. Qual Health Res 2015;25(9):1212–1222. DOI: 10.1177/1049732315588501. 10.1177/1049732315588501 26184336

[34] Morse JM (2000). Determining sample size.

[35] Burnard P, Gill P, Stewart K, Treasure E, Chadwick B (2008). Analysing and presenting qualitative data. Br Dent J.

[36] Grove SK, Burns N, Gray JR. Understanding nursing research: building an evidence-based practice: Elsevier Health Sciences; 2014.

[37] Braun V, Clarke V (2006). Using thematic analysis in psychology. Qual Res Psychol.

[38] Erlandsson K, Häggström-Nordin E (2010). Prenatal parental education from the perspective of fathers with experience as primary caregiver immediately following birth: a phenomenographic study. The J J Perinat Educ.

[39] Malata A. Development of childbirth education for Malawian women 2004. 10.1111/j.1365-2648.2007.04380.x 17824941

[40] Yidana A, Ziblim S-D, Yamusah B (2018). Male partner involvement in birth preparedness and utilization of antenatal care services: a study in the West Mamprusi municipality of northern Ghana. World J Public Health.

[41] August F, Pembe AB, Mpembeni R, Axemo P, Darj E (2015). Men’s knowledge of obstetric danger signs, birth preparedness and complication readiness in rural Tanzania. PLoS One.

[42] Lori JR, Dahlem CHY, Ackah JV, Adanu RMK (2014). Examining antenatal health literacy in Ghana. Journal of nursing Scholaship. J Nurs Scholarsh.

[43] Weldearegay HG (2015). Determinant factors of male involvement in birth preparedness and complication readiness at Mekelle town; a community based study. Sci J Public Health.

[44] Tadesse M, Boltena AT, Asamoah BO (2018). Husbands' participation in birth preparedness and complication readiness and associated factors in Wolaita Sodo town, southern Ethiopia. Afr j prim health care fam med.

[45] Tobin EA, Ofili AN, Enebeli N, Enueze O (2014). Assessment of birth preparedness and complication readiness among pregnant women attending primary health care Centres in Edo state, Nigeria. Ann Niger Med.

[46] Kaye DK, Kakaire O, Nakimuli A, Osinde MO, Mbalinda SN, Kakande N (2014). Male involvement during pregnancy and childbirth: men's perceptions, practices and experiences during the care for women who developed childbirth complications in Mulago hospital, Uganda. BMC Pregnancy Childbirth..

[47] Davis J, Vyankandondera J, Luchters S, Simon D, Holmes W (2016). Male involvement in reproductive, maternal and child health: a qualitative study of policymaker and practitioner perspectives in the Pacific. Reprod Health.

[48] Brown A, Davies R (2014). Fathers' experiences of supporting breastfeeding: challenges for breastfeeding promotion and education. Matern Child Nutr.

[49] Duggan R, Adejumo O (2012). Adolescent clients’ perceptions of maternity care in KwaZulu-Natal, South Africa. Women Birth.

[50] Opondo C, Redshaw M, Savage-McGlynn E, Quigley MA (2016). Father involvement in early child-rearing and behavioural outcomes in their pre-adolescent children: evidence from the ALSPAC UK birth cohort. BMJ Open.

[51] Mohlahla BKF, Gregson S, Boily MC (2012). Barriers to involvement of men in ANC and VCT in Khayelitsha, South Africa. AIDS care.

[52] Mphonda SM, Rosenberg NE, Kamanga E, Mofolo I, Mwale G, Boa E (2014). Assessment of peer-based and structural strategies for increasing male participation in an antenatal setting in Lilongwe. Malawi Afr J Reprod Health.

[53] Nyondo AL, Chimwaza AF, Muula AS. Exploring the relevance of male involvement in the prevention of mother to child transmission of HIV services in Blantyre. Malawi BMC Int Health Hum Rights. 2014;14(30). 10.1186/s12914-014-0030-y. 10.1186/s12914-014-0030-y PMC4422229 25359447

[54] Pauleta JR, Pereira NM, Graça LM (2010). Sexuality during pregnancy. Sex Med.

[55] Polomeno V. Marriage in the transition to parenthood: how can perinatal education help? Or can it? Int J Childbirth Edu. 2007;22(2). https://www.thefreelibrary.com.

[56] Polomeno V. The teaching of conjugal vulnerability during the transition to parenthood. Int J Childbirth Educ. 2014;29(1). https://www.thefreelibrary.com.

[57] Nyondo AL, Chimwaza AF, Muula AS (2014). Stakeholders’ perceptions on factors influencing male involvement in prevention of mother to child transmission of HIV services in Blantyre. Malawi BMC Public Health.

[58] Larsson EC, Thorson A, Nsabagasani X, Namusoko S, Popenoe R, Ekström AM (2010). Mistrust in marriage-reasons why men do not accept couple HIV testing during antenatal care-a qualitative study in eastern Uganda. BMC Public Health.

[59] Olawole-Isaac A, Oni G, Oladosun M, Muyiwa A. Gender-based violence and pregnancy outcomes among couples and cohabiting Partners in Nigeria. 2016. URI: http://eprints.covenantuniversity.edu.ng/id/eprint/6636

[60] Turan JM, Nalbant H, Bulut A, Sahip Y (2001). Including expectant fathers in antenatal education programmes in Istanbul, Turkey. Reprod Health Matters.

[61] Svensson J, Barclay L, Cooke M (2006). The concerns and interests of expectant and new parents: assessing learning needs. J Perinat Educ.

[62] Bäckström C, Hertfelt Wahn E (2011). Support during labour: first-time fathers’ descriptions of requested and received support during the birth of their child. Midwifery.

[63] Kululanga LI, Malata A, Chirwa E, Sundby J (2012). Malawian fathers' views and experiences of attending the birth of their children: a qualitative study. BMC Pregnancy Childbirth.

[64] Naim M, Bhutto E (2000). Sexuality during pregnancy in Pakistani women. J Pak Med Assoc.

[65] Williamson M, McVeigh C, Baafi M (2008). An Australian perspective of fatherhood and sexuality. Midwifery.

[66] Malawi Government. Sexual Reproductive Health and Right Policy. Lilongwe, Malawi 2011.

[67] Redshaw M, Henderson J (2013). Fathers’ engagement in pregnancy and childbirth: evidence from a national survey. BMC Pregnancy Childbirth..

[68] Simbar M, Nahidi F, Tehrani R, Akbarzadeh A (2011). Educational needs assessment for men's participation in perinatal care. East Mediterr Health J.

[69] Soliman M (1999). Impact of antenatal counselling on couples' knowledge and practice of contraception in Mansoura, Egypt.

[70] Kabagenyi A, Jennings L, Reid A, Nalwadda G, Ntozi J, Atuyambe L (2014). Barriers to male involvement in contraceptive uptake and reproductive health services: a qualitative study of men and women’s perceptions in two rural districts in Uganda. Reprod Health.

[71] Noronha JA, Bhaduri A, Bhat HV, Kamath A (2013). Interventional study to strengthen the health promoting behaviours of pregnant women to prevent anaemia in southern India. Midwifery.

