# Supplementary material for: Exploring antenatal education content for couples in Blantyre, Malawi
Source: BMC Pregnancy Childbirth. 2018 Dec 17;18:497. doi: 10.1186/s12884-018-2137-y (PMC6296087; doi:10.1186/s12884-018-2137-y)
Supplement: Supplementary file 1 — RATS checklist. (DOCX 24 kb) [file 12884_2018_2137_MOESM1_ESM.docx]

| **ASK THIS OF THE MANUSCRIPT** | **THIS SHOULD BE INCLUDED IN THE MANUSCRIPT** | **Reference in manuscript** |
| --- | --- | --- |
| **R Relevance of study question** |  |  |
| Is the research question interesting? | Research question explicitly stated | The research question is explicitly stated towards the end of the introduction section (pg. 4). |
| Is the research question relevant to clinical practice, public health, or policy? | Research question justified and linked to the existing knowledge base (empirical research, theory, policy) | The introduction section has shown what is existing in the area of study and has justified the research question (pgs.3-5). |
| **A Appropriateness of qualitative method** |  |  |
| Is qualitative methodology the best approach for the study aims?   - *Interviews:* experience, perceptions, behaviour, practice, process - *Focus groups:* group dynamics, convenience, non-sensitive topics - *Ethnography:* culture, organizational behaviour, interaction - *Textual analysis:* documents, art, representations, conversations | Study design described and justified i.e., why was a particular method (e.g., interviews) chosen? | The study design and the rationale for using Focus Group Discussions, In depth interviews and Key Informant Interviews is justified (pg. 5). |
| **T Transparency of procedures** |  |  |
| *Sampling* |  |  |
| Are the participants selected the most appropriate to provide access to the type of knowledge sought by the study?  Is the sampling strategy appropriate? | Criteria for selecting the study sample justified and explained   - *theoretical:* based on preconceived or emergent theory - *purposive:* diversity of opinion - *volunteer:* feasibility, hard-to-reach groups | Key Informants, participants for in depth interviews and male participants for FGDs were purposively selected while female FGDs participants were conveniently sampled. The criteria and justification are on pages 7 -11 |
| *Recruitment* |  |  |
| Was recruitment conducted using appropriate methods? | Details of how recruitment was conducted and by whom | The Principal Investigator (PI) and the research assistant recruited the pregnant women for focus group discussions at the antenatal clinic. Male participants for focus group discussions, couples and men who had escorted their wives previously to the antenatal clinic were recruited from the outpatient department and the catchment areas of Mpemba and South Lunzu health centres. Key informants (KIs) were selected based on their roles and responsibilities in various institutions, which were both health related and non-health related. See page (7-11) |
| *Is the sampling strategy appropriate?* |  |  |
| Could there be selection bias? | Details of who chose not to participate and why | None of the participants refused participation except two male participants for FGDs and they did not give reasons for their refusal. (see page 8 -9). |
| *Data collection* |  |  |
| Was collection of data systematic and comprehensive? | Method(s) outlined and examples given (e.g., interview questions) | The methods followed in the study are outlined on pages 11-13. The interview guides are appended as additional files |
| Are characteristics of the study group and setting clear? | Study group and setting clearly described | The study group and setting are clearly described on pages 5-11 |
| Why and when was data collection stopped, and is this reasonable? | End of data collection justified and described | The period of data collection and the reason for stopping data collection is on pg5. |
| *Role of researchers* |  |  |
| Is the researcher(s) appropriate? How might they bias (good and bad) the conduct of the study and results? | Do the researchers occupy dual roles (clinician and researcher)? Are the ethics of this discussed? Do the researcher(s) critically examine their own influence on the formulation of the research question, data collection, and interpretation? | The researchers had no dual roles, see page 35. The ethical considerations in the study are described on page 34. The interpretation of the results is backed by participants quotes in the results section ( pgs. 18-28) |
| *Ethics* |  |  |
| Was informed consent sought and granted? | Informed consent process explicitly and clearly detailed | Informed consent procedures described under the Methods section on pages 11-13. |
| Were participants’ anonymity and confidentiality ensured? | Anonymity and confidentiality discussed | All quotes were anonymised. Data is stored securely in locked cabinets at the Malawi College of Medicine. Identifiers were removed from the scripts.(see pages 13 – 14) on Data management section |
| Was approval from an appropriate ethics committee received? | Ethics approval cited | Ethical approval was granted and is cited on page 34. |
| **S Soundness of interpretive approach** |  |  |
| *Analysis* |  |  |
| Is the type of analysis appropriate for the type of study?   - *thematic:* exploratory, descriptive, hypothesis generating - *framework:* e.g., policy - *constant comparison/grounded theory:* theory generating, analytical | Analytic approach described in depth and justified  *Indicators of quality:* Description of how themes were derived from the data (inductive or deductive)  Evidence of alternative explanations being sought  Analysis and presentation of negative or deviant cases | The data analysis approach is detailed on pages 14 under data analysis. |
| *Are the interpretations clearly presented and adequately supported by the evidence?* |  |  |
| Are quotes used and are these appropriate and effective? | Description of the basis on which quotes were chosen  Semi-quantification when appropriate  Illumination of context and/or meaning, richly detailed | Quotes presented under results (pg 18-28) and also as in the appended document that shows variation in responses among the participants. |
| Was trustworthiness/reliability of the data and interpretations checked? | Method of reliability check described and justified e.g., was an audit trail, triangulation, or member checking employed? Did an independent analyst review data and contest themes? How were disagreements resolved? | Triangulation by data source and collection methods was employed (see page 5 ) Additionally we employed constant comparison method during coding; codes were validated by an independent qualitative researcher). After each interview and discussion verification of what was discussed was achieved through member checking (see pages 11 – 15) |
| *Discussion and presentation* |  |  |
| Are findings sufficiently grounded in a theoretical or conceptual framework?  Is adequate account taken of previous knowledge and how the findings add? | Findings presented with reference to existing theoretical and empirical literature, and how they contribute | The results are grounded in literature. Refer to pages 24-28 where we have discussed our results in contrast and comparison with previous research in the area. |
| Are the limitations thoughtfully considered? | Strengths and limitations explicitly described and discussed | Strength and limitations of the study have been reported ( see page 33) |
| Is the manuscript well written and accessible? | Evidence of following guidelines (format, word count)  Detail of methods or additional quotes contained in appendix  Written for a health sciences audience | The RATS checklist is included as additional file |
| Are red flags present? These are common features of ill-conceived or poorly executed qualitative studies, are a cause for concern, and must be viewed critically. They might be fatal flaws, or they may result from lack of detail or clarity. | *Grounded theory:* not a simple content analysis but a complex, sociological, theory generating approach  *Jargon:* descriptions that are trite, pat or jargon filled should be viewed sceptically  *Over interpretation:* interpretation must be grounded in "accounts" and semi-quantified if possible or appropriate  *Seems anecdotal, self evident:* may be a superficial analysis, not rooted in conceptual framework or linked to previous knowledge, and lacking depth  *Consent process thinly discussed:* may not have met ethics requirements  *Doctor-researcher:* consider the ethical implications for patients and the bias in data collection and interpretation | None |

*The RATS guidelines modified for BioMed Central are copyright Jocalyn Clark. They can be found in Clark JP:*How to peer review a qualitative manuscript*. In*Peer Review in Health Sciences*. Second edition. Edited by Godlee F, Jefferson T. London: BMJ Books; 2003:219-235*
