# Supplementary material for: Exploring antenatal education content for couples in Blantyre, Malawi
Source: BMC Pregnancy Childbirth. 2018 Dec 17;18:497. doi: 10.1186/s12884-018-2137-y (PMC6296087; doi:10.1186/s12884-018-2137-y)
Supplement: Supplementary file 2 — A guide for focus group discussion. (DOCX 17 kb) [file 12884_2018_2137_MOESM2_ESM.docx]

**ADDITIONAL FILE 2: FOCUS GROUP DISCUSSION GUIDE**

**Introduction**

I would like to thank you for accepting to take part in this discussion. My name is Maria Chifuniro Chikalipo, a student at College of Medicine in Blantyre. I am conducting these discussions with groups of young (18 to 25 years old) and older (26 years and above) men and women from this catchment area. The topic of discussion during these discussions is male partners’ involvement in antenatal education. When we talk of male involvement in antenatal education we are looking at how male partners are engaged in antenatal education with their spouses focusing on all the activities which occur during antenatal education such as messages given and mode of delivery which includes singing and clapping of hands. The information gathered will assist in designing a package which will be used to teach couples during antenatal education. We feel that when male partners are involved in antenatal education sessions with their spouses the health of the mother and the whole family is likely to improve as the education will enable the couple to prepare for birth and emergencies associated with childbirth timely. Your opinions will be greatly valued.

**Informed consent section**

Before we start the discussions I would like to ask for your written informed consent with the understanding that you have read the participant information sheet and have understood the details of the study.

*Note: Informed consent was obtained after participants had read the participant’s information sheet which contained details about the study. P*articipant’s social demographic information was collected according to the socio demographic questionnaire.

**Discussion**

Before we start the discussions can you tell me what safe motherhood mean to us?

**Objective one: Identification of factors that can influence male partners to visit the antenatal clinic with their spouses for antenatal education sessions.**

1. What is the magnitude of male partner involvement in antenatal care in this area?
2. What is the relevance of male partners’ participation in antenatal education with their spouses
3. What are some of the factors that can motivate male partners to attend together with their spouses’ antenatal education sessions?

Probe for personal, facility and social cultural factors and how they can facilitate male partner participation in antenatal education

Probe for availability of policy/guidelines which facilitate male involvement in maternity care

Probe for amount of revenue lost when a male partner accompanies a spouse for antenatal care

Probe for how time lost in escorting a wife to the clinic is compensated

1. What are some of the factors that can discourage male partners to attend together with their spouses’ antenatal education sessions?

Probe for personal, facility and social cultural factors including religious beliefs and how they can hinder male partner participation in antenatal education

Probe for personal, facility and social cultural factors and how they can hinder male partner participation in antenatal education

**Objective two: assessment of relevant content for couple antenatal education**

1. How do men and women get information related to childbirth?
2. Among the sources of information people get information from, can you please explain the most reliable source with reasons
3. What type of information should be discussed with expectant couples during antenatal education sessions?

Probe on information needed related to pregnancy

Probe on information needed related to labour and delivery

Probe on information needed related to post-partum period

Probe on type of messages given to expectant couples culturally

Probe on how culture/religion influence choice of topic for couple antenatal education

**Objective three: Assessment of organization of couple antenatal education.**

1. In the communities, can we explain on how childbirth information is delivered to couples?

Probe on who gives the information

Probe on the period (gestation) the education is given to the expectant couples

1. How should couple antenatal education be designed?

Probe on how husbands should be invited for the education

Probe on number of sessions a male partner should attend the education with the wife

Probe on the sex and professional background of the facilitator for couple education

Probe on how it should be delivered: group versus individual, men and women separate
