# Supplementary material for: Exploring antenatal education content for couples in Blantyre, Malawi
Source: BMC Pregnancy Childbirth. 2018 Dec 17;18:497. doi: 10.1186/s12884-018-2137-y (PMC6296087; doi:10.1186/s12884-018-2137-y)
Supplement: Supplementary file 5 — Distribution of subthemes. (DOCX 18 kb) [file 12884_2018_2137_MOESM5_ESM.docx]

**Additional File 5: Distribution of Sub themes on content for couple antenatal education among men, women, nurse/midwives, couples and men who have been to antenatal clinic**

| **Theme:**  **information for couple antenatal education service** | **Men** | **Women** | **Nurse/midwives** | **Couples** | **KII** | **Men who have been to clinic** | **Health Care Workers** |
| --- | --- | --- | --- | --- | --- | --- | --- |
| 1. **Antepartum education needs** |  |  |  |  |  |  |  |
| Description of pregnancy | + | - | + | + | + | - | * |
| Care of a pregnant woman | + | + | + | + | + | + | - |
| Role of men during perinatal period | + | - | - | - | + | + | * |
| Family life |  |  |  |  | + | + | * |
| Birth preparedness and complication readiness plan | + | + | + | + | + | + |  |
| Prevention of mother to child HIV transmission and syphilis testing | + | - | + | + | + | + |  |
| Sexual activity during pregnancy | + | - | + | + | + | + |  |
|  |  |  |  |  |  |  |  |
| 1. **Intrapartum education needs** |  |  |  |  |  |  |  |
| Signs of labour | - | + | + | + | - | + | * |
| Giving birth | + | + | + | + | + | + | - |
|  |  |  |  |  |  |  |  |
| 1. **Postpartum education needs** |  |  |  |  |  |  |  |
| Sex after delivery | + | - | + | + | + | + | * |
| Baby care | + | + | - | - | - | - | * |
| Danger signs of a baby | - | - | - | + | - | - |  |
| Family planning | + | + | + | + | + | + |  |
|  |  |  |  |  |  |  |  |

**Key + = Mentioned as a topic - = Not mentioned as a topic**
